# Insufficient adenosine-induced hyperemia is a major determinant of discordance between non-hyperemic pressure ratio and fractional flow reserve

**DOI:** 10.1038/s41598-023-27929-1

**Published:** 2023-01-13

**Authors:** Hidenari Matsumoto, Satoshi Higuchi, Hideaki Tanaka, Ryota Masaki, Seita Kondo, Hiroaki Tsujita, Toshiro Shinke

**Affiliations:** Division of Cardiology, Showa University School of Medicine, 1-5-8 Hatanodai, Shinagawa-ku, Tokyo, 142-8555 Japan

**Keywords:** Cardiology, Medical research

## Abstract

Adenosine occasionally overestimates fractional flow reserve (FFR) values (i.e., insufficient adenosine-induced hyperemia), leading to low non-hyperemic pressure ratios (NHPR)–high FFR discordance. We investigated the impact of insufficient adenosine-induced hyperemia on NHPR–FFR discordance and the reclassification of functional significance. We measured resting distal-to-aortic pressure ratio (Pd/Pa) and FFR by using adenosine (FFR_ADN_) and papaverine (FFR_PAP_) in 326 patients (326 vessels). FFR_ADN_ overestimation was calculated as FFR_ADN_ − FFR_PAP_. We explored determinants of low Pd/Pa − high FFR_ADN_ discordance (Pd/Pa ≤ 0.92 and FFR_ADN_ > 0.80) versus high Pd/Pa − low FFR_ADN_ discordance (Pd/Pa > 0.92 and FFR_ADN_ ≤ 0.80). Reclassification of functional significance was defined as FFR_ADN_ > 0.80 and FFR_PAP_ ≤ 0.80. Multivariable analysis identified FFR_ADN_ overestimation (p = 0.002) and heart rate at baseline (p = 0.048) as independent determinants of the low Pd/Pa–high FFR_ADN_ discordance. In the low Pd/Pa–high FFR_ADN_ group (n = 26), papaverine produced a further decline in the FFR value in 21 vessels (81%) compared with FFR_ADN_, and the reclassification was observed in 17 vessels (65%). Insufficient adenosine-induced hyperemia is a major determinant of the low resting Pd/Pa–high FFR discordance. Physicians should bear in mind that the presence of low NHPR–high FFR discordance may indicate a false-negative FFR result.

## Introduction

Measuring fractional flow reserve (FFR) has been a standard method for guiding coronary revascularization in chronic coronary syndrome^1–4^. To simplify physiological assessment procedures, non-hyperemic pressure ratios (NHPRs) without the need for a vasodilator were introduced^5^. Resting distal-to-aortic pressure ratio (Pd/Pa) during the entire cardiac cycle is available with the use of any pressure-monitoring system, providing a universal resting physiological metric. Although recent clinical guidelines recommend NHPRs as well as FFR for the selection of revascularization strategies^6–8^, one-fifth of cases demonstrate discordance of the physiological significance between NHPRs and FFR^9–13^. Since the principle of FFR is based on maximal hyperemia^3,14^, insufficient hyperemia (i.e. an overestimation of FFR) is a potential cause of discordance with a low NHPR and a high FFR^5^.

It has been demonstrated that intravenous adenosine, the vasodilator that is most commonly used for hyperemia induction^1–4,14^, occasionally fails to induce maximal hyperemia compared to other hyperemic stimuli, such as papaverine^15–18^. If low NHPR–high FFR discordance is associated with insufficient adenosine-induced hyperemia, vessels that have low NHPR–high FFR discordance may show positive FFR results when another stimulus is used, providing a false-negative result based on an adenosine-induced FFR. Conversely, high NHPR–low FFR discordance may indicate sufficient adenosine-induced hyperemia. The impact of insufficient adenosine-induced hyperemia on NHPR–FFR discordance has not been investigated. Earlier studies used only adenosine for hyperemia induction, thereby precluding an assessment of adenosine’s role in NHPR–FFR discordance^19–22^.

Adenosine produces hyperemia through adenosine A_2a_ receptors in vascular smooth muscles^4^, whereas papaverine induces maximal hyperemia most reliably by causing a direct relaxation of the vascular smooth muscle^23^. In our present investigation, patients’ FFR values were measured using adenosine (FFR_ADN_) and papaverine (FFR_PAP_). FFR_PAP_ was used as a reference standard of functional significance. We sought to determine the impact of insufficient adenosine-induced hyperemia on resting Pd/Pa–FFR_ADN_ discordance and the reclassification of functional significance.

## Methods

### Study patients

This retrospective study included 365 patients with chronic coronary syndrome who underwent an FFR assessment for standard clinical indications. If a patient required FFR assessments for two or more vessels, only the first vessel was included in this study. All of the patients were asked to abstain from food and beverages for > 3 h before the catheterization. More prolonged caffeine abstinence was left to the physician’s discretion. The exclusion criteria consisted of any contraindications for adenosine or papaverine, patients with severe arrhythmia (e.g., frequent ectopic beats or atrial fibrillation), the presence of significant valvular disease, an ostial lesion, a prior coronary artery bypass graft, and the use of a theophylline-containing medication. Patients with insufficient pressure data quality, including a signal drift value of more than ± 0.03 after the pullback of the pressure wire and inadequate waveform tracings, were also excluded.

The coronary physiology assessment was performed as part of the routine diagnostic coronary angiography procedures for clinical purposes. All methods were performed in accordance with the relevant guidelines and regulations. Written informed consent for the invasive physiology assessment was obtained from all of the patients before the procedure. The Institutional Review Board approved this retrospective study (reference #3234/ Showa University School of Medicine; 31 August, 2021) and waived the requirement of patient approval for the use of patient data and medical records for research.

### Coronary physiologic measurements

Coronary angiography was performed in a standard manner for each patient. Intracoronary isosorbide dinitrate (2 mg) was administered before the physiological assessments. With the use of a coronary-pressure guidewire (Philips Volcano or Abbott Vascular) and a 5- or 6-F guiding catheter without side holes, the distal coronary pressure (Pd) and the aortic pressure (Pa) were obtained simultaneously. The patient’s resting Pd/Pa ratio was recorded after his/her full recovery from the influence of contrast media, isosorbide dinitrate, or saline flush.

Adenosine was administered continuously via a femoral vein or a large forearm vein at 140 μg/kg/min for > 150 s^3,4,14,24^. In cases in which steady-state hyperemia was not achieved during the adenosine infusion, the infusion was continued for a minimum of 180 s. Papaverine was used as the last agent to obtain a reliable pull-back curve, as it induces hyperemia with minimal variations in Pd/Pa^25^. After confirming that Pd/Pa values had returned to the baseline level, with an interval of ≥ 5 min after the termination of adenosine infusion, intracoronary papaverine (8–10 mg in the right coronary artery or 12–15 mg in the left coronary artery) was given through the coronary catheter, followed by 5 mL of saline^14,26^. Approximately 20 s after the papaverine injection, an FFR pullback recording was performed manually, and the presence of pressure-wire drift was checked.

### Data analysis

#### Resting Pd/Pa and FFR

Experienced observers blinded to the patients’ coronary angiography results and clinical data manually reviewed the pressure recordings. Pressure waveforms from ectopic beats and the adjacent beats were not included in the analysis. Resting Pd/Pa ratio was calculated as the mean Pd to the mean Pa, and ≤ 0.92 was regarded as a positive ratio^5,11^. FFR_ADN_ was measured during the steady-state hyperemic plateau phase > 60 s after the initiation of the adenosine infusion and > 15 s after the transition to hyperemia^18,27^. The lowest Pd/Pa values on a beat-to-beat basis for adenosine and papaverine were regarded as FFR_ADN_ and FFR_PAP_, respectively^15–18,28^, and ≤ 0.80 was used as the cut-off for FFR_ADN_ and FFR_PAP_^1–3,29^. The difference in FFR values between adenosine and papaverine was calculated as FFR_ADN_ − FFR_PAP_^18^. The reclassification of functional significance was defined as FFR_ADN_ > 0.80 and FFR_PAP_ ≤ 0.80 (false-negative by adenosine), and reverse reclassification was defined as FFR_ADN_ ≤ 0.80 and FFR_PAP_ > 0.80 (false-positive by adenosine). Based on the FFR pull-back curve, the physiological pattern of disease was classified as focal, diffuse, or a combination of both (mixed) by the consensus of experienced observers^30^.

We classified the enrolled vessels into four groups according to their resting Pd/Pa and FFR_ADN_ values: (i) high resting Pd/Pa–high FFR_ADN_ (resting Pd/Pa > 0.92 and FFR_ADN_ > 0.80), (ii) high resting Pd/Pa–low FFR_ADN_ (resting Pd/Pa > 0.92 and FFR_ADN_ ≤ 0.80), (iii) low resting Pd/Pa–high FFR_ADN_ (resting Pd/Pa ≤ 0.92 and FFR_ADN_ > 0.80), and (iv) low resting Pd/Pa–low FFR_ADN_ (resting Pd/Pa ≤ 0.92 and FFR_ADN_ ≤ 0.80). We evaluated the clinical and pathophysiological characteristics between the vessels with low resting Pd/Pa–high FFR_ADN_ discordance and the vessels with high Pd/Pa–low FFR_ADN_ discordance, based on a study of NHPR–FFR discordance^22^.

#### Coronary angiography

Quantitative coronary angiography was performed in optimal projections with a commercially available system (CAAS Workstation version 7.5, Pie Medical Imaging) by independent investigators blinded to the physiological results and clinical data. The reference diameter, minimum lumen diameter, and lesion length were measured by using the external diameter of the catheter as a scaling device, and the diameter stenosis was calculated.

### Statistical analysis

Continuous variables were presented as medians with interquartile ranges (IQRs). Categorical variables were presented as numbers and proportions. Comparisons between adenosine and papaverine were done with the Wilcoxon signed-rank test for quantitative variables and with the McNemar test for categorical variables. Correlations between two variables were assessed with Spearman’s rank correlation coefficient. Between-group comparisons were made with the unpaired-samples t-test or the Mann–Whitney U-test for quantitative variables and with the χ^2^ test or Fisher’s exact test for categorical variables, as appropriate. Multivariable logistic regression analysis was performed to determine factors associated with the low resting Pd/Pa–high FFR discordance versus the high resting Pd/Pa–low FFR discordance. Clinical, angiographic, and hemodynamic parameters with a univariable association of p < 0.10 and FFR_ADN_ − FFR_PAP_ were included in the multivariable model. The results were presented as the odds ratio and 95% confidence interval. Statistical analyses were performed using JMP® Pro, ver. 16.0.0 (SAS, Cary, NC). A p-value < 0.05 was considered significant.

## Results

### Procedures

Among the 365 vessels in the 365 patients, 39 were eliminated from the analysis because of difficulty in advancing the pressure wire far distal to the index lesion (n = 6), side effects from adenosine (n = 8) or papaverine (n = 2), sensor drift (n = 13), or insufficient waveform tracings (n = 10). A final total of 326 vessels were included in the study. The patient and lesion characteristics are summarized in Table 1.

**Table 1:** Patient and lesion characteristics.

No. of patients	326
Age, years	72 (65–78)
Male, n (%)	252 (77%)
Hypertension, n (%)	232 (71%)
Diabetes mellitus, n (%)	133 (41%)
Dyslipidemia, n (%)	235 (72%)
Current smoker	65 (20%)
eGFR, mL/min	64.6 (52.2–75.2)
eGFR < 60 mL/min, n (%)	123 (38%)
Hemodialysis, n (%)	25 (8%)
Hemoglobin, g/dL	13.5 (12.1–14.5)
Prior myocardial infarction, n (%)	79 (24%)
Prior revascularization, n (%)	127 (39%)
Multivessel disease, n (%)	179 (55%)
Quantitative coronary angiography	
Reference diameter, mm	2.8 (2.4–3.3)
Minimal luminal diameter, mm	1.4 (1.1–1.7)
Diameter stenosis, %	50.6 (42.4–57.5)
Lesion length, mm	11.5 (8.1–16.4)
Hemodynamic parameters	
Heart rate at baseline, beats/min	67 (61–74)
Pa at baseline, mmHg	91 (82–101)
Pd/Pa ratio at baseline	0.93 (0.88–0.96)
FFR_ADN_	0.79 (0.73–0.86)
FFR_PAP_	0.77 (0.70–0.84)

Values are expressed as medians (interquartile ranges) or numbers (percentages).

*eGFR* estimated glomerular rate, *FFR*_*ADN*_ fractional flow reserve value associated with adenosine, *FFR*_*PAP*_ fractional flow reserve value associated with papaverine, *LAD* left anterior descending coronary artery, *LM* left main coronary artery, *Pa* mean aortic pressure, *Pd* mean distal coronary pressure.

### Resting Pd/Pa and FFR

**Figure 1:**
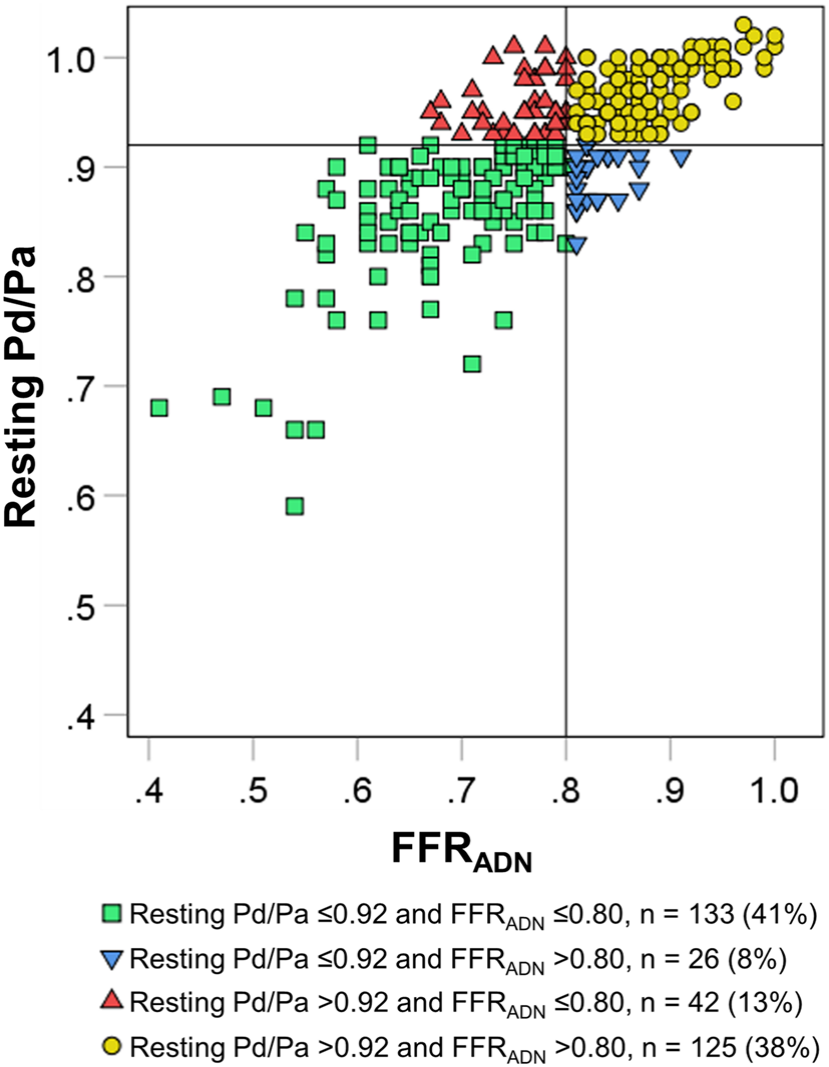
Scatter plot of resting Pd/Pa and FFR_ADN_. The vessels were classified into four groups by using the predefined cut-off values of 0.92 for resting Pd/Pa and 0.80 for FFR_ADN_. Among 326 vessels, 68 (21%) demonstrated discordance: low resting Pd/Pa–high FFR_ADN_ in 26 vessels (8%) and high Pd/Pa–low FFR_ADN_ in 42 vessels (13%). *FFR*_*ADN*_ fractional flow reserve value associated with adenosine, *Pd/Pa* distal-to-aortic pressure ratio.

The median resting Pd/Pa was 0.93 (IQR 0.88–0.96); the median FFR_ADN_ was 0.79 (IQR 0.73–0.86), and the median FFR_PAP_ was 0.77 (IQR 0.70–0.84). Figure 1 is a scatterplot of resting Pd/Pa and FFR_ADN_ values: there was a moderate correlation between these two indices (ρ = 0.756, p < 0.001).

Using the predefined cutoff values of resting Pd/Pa and FFR_ADN_, we observed that 258 vessels (79%) had concordant results, which consisted of the low resting Pd/Pa–low FFR in 133 vessels (41%) and the high resting Pd/Pa–high FFR in the other 125 (38%). The remaining 68 vessels (21%) demonstrated discordant results and were comprised of 26 vessels (8%) with the low resting Pd/Pa–high FFR_ADN_ and 42 vessels (13%) with the high Pd/Pa–low FFR_ADN_.

**Figure 2:**
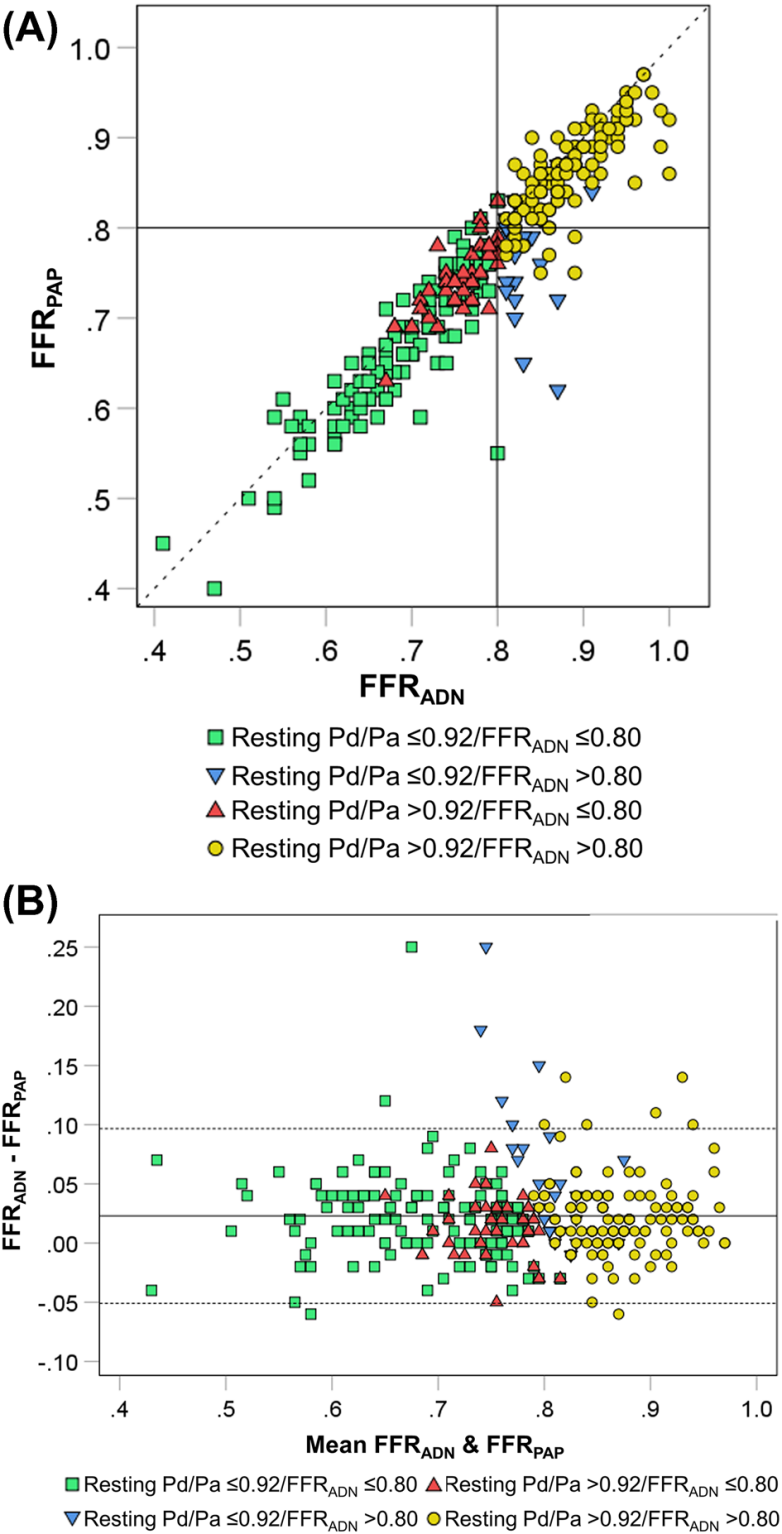
Comparison of FFR_ADN_ and FFR_PAP_. (**A**) Scatter plot. FFR_ADN_ and FFR_PAP_ were highly correlated (ρ = 0.926, p < 0.001). *FFR*_*ADN*_ fractional flow reserve value associated with adenosine, *FFR*_*PAP*_ fractional flow reserve value associated with papaverine. (**B**) Bland–Altman plot. There was a positive bias towards the overestimation of FFR by adenosine (p < 0.001), with the mean difference of 0.02 and the 95% limits of agreements of − 0.05 and 0.10.

Figure 2 compares FFR_ADN_ and FFR_PAP_ values. As shown in a scatterplot (Fig. 2A), FFR_ADN_ and FFR_PAP_ values were highly correlated (ρ = 0.926, p < 0.001). Bland–Altman analysis (Fig. 2B) revealed a significant bias toward the overestimation of FFR by adenosine (p < 0.001), with the mean difference of 0.02 and the 95% limits of agreements of −0.05 and 0.10.

### Comparison of the two discordant groups

Table 2 summarizes the patient and lesion characteristics in the low Pd/Pa–high FFR_ADN_ and high Pd/Pa–low FFR_ADN_ groups. Diabetes mellitus was significantly more frequent in the low Pd/Pa–high FFR_ADN_ group compared to the high Pd/Pa–low FFR_ADN_ group: 50% (13/26) vs. 26% (11/42), p = 0.046. The low Pd/Pa–high FFR_ADN_ group tended to receive hemodialysis more frequently: 6% (4/26) vs. 2% (1/42), p = 0.067. The LAD location, the quantitative coronary angiography parameters, and the physiological pattern did not differ between the two groups. Heart rate at baseline was significantly higher in the low Pd/Pa–high FFR_ADN_ group compared to the high Pd/Pa–low FFR_ADN_ group: 73 (IQR 61–81) vs. 62 (IQR 57–71), p = 0.008.

**Table 2:** Comparison between two discordant groups.

	Pd/Pa ≤ 0.92	Pd/Pa > 0.92	p value
	FFR_ADN_ > 0.80	FFR_ADN_ ≤ 0.80	
	(n = 26)	(n = 42)	
Age, years	73 (66–79)	69 (64–76)	0.103
Male, n (%)	19 (73%)	35 (83%)	0.309
Hypertension, n (%)	16 (62%)	25 (60%)	0.869
Diabetes mellitus, n (%)	13 (50%)	11 (26%)	0.046
Dyslipidemia, n (%)	18 (69%)	28 (67%)	0.826
Current smoker	6 (23%)	8 (19%)	0.690
eGFR, mL/min	67.1 (48.1–78.5)	64.2 (56.2–71.4)	0.767
eGFR < 60 mL/min, n (%)	15 (58%)	27 (64%)	0.587
Hemodialysis, n (%)	4 (6%)	1 (2%)	0.067
Hemoglobin, g/dL	13.6 (12.8–14.8)	13.8 (12.4–14.9)	0.754
Prior myocardial infarction, n (%)	5 (19%)	9 (21%)	0.828
Prior revascularization, n (%)	10 (38%)	19 (45%)	0.583
LAD location, n (%)	13 (50%)	22 (52%)	0.849
Multivessel disease, n (%)	17 (65%)	27 (64%)	0.927
Quantitative coronary angiography			
Reference diameter, mm	2.6 (2.5–3.0)	2.9 (2.4–3.4)	0.398
Minimal luminal diameter, mm	1.3 (1.1–1.7)	1.4 (1.0–1.7)	0.883
Diameter stenosis, %	52.5 (41.6–57.6)	51.0 (44.8–57.9)	0.762
Lesion length, mm	10.2 (6.6–16.6)	11.3 (9.3–15.2)	0.659
Physiological pattern			
Focal/mixed/diffuse, n (%)	9/6/11	18/9/15	0.788
Hemodynamic parameters			
Heart rate at baseline, beats/min	73 (61–81)	62 (57–71)	0.008
Pa at baseline, mmHg	90 (77–102)	87 (82–100)	0.499
Pd/Pa ratio at baseline	0.90 (0.87–0.91)	0.95 (0.94–0.98)	N/A
FFR_ADN_	0.82 (0.81–0.84)	0.77 (0.74–0.79)	N/A
FFR_PAP_	0.79 (0.74–0.81)	0.75 (0.72–0.78)	N/A

Values are expressed as medians (interquartile ranges) or numbers (percentages).

*eGFR* estimated glomerular rate, *FFR*_*ADN*_ fractional flow reserve value associated with adenosine, *FFR*_*PAP*_ fractional flow reserve value associated with papaverine, *LAD* left anterior descending coronary artery, *N/A* not applicable, *Pa* mean aortic pressure, *Pd* mean distal coronary pressure.

**Figure 3:**
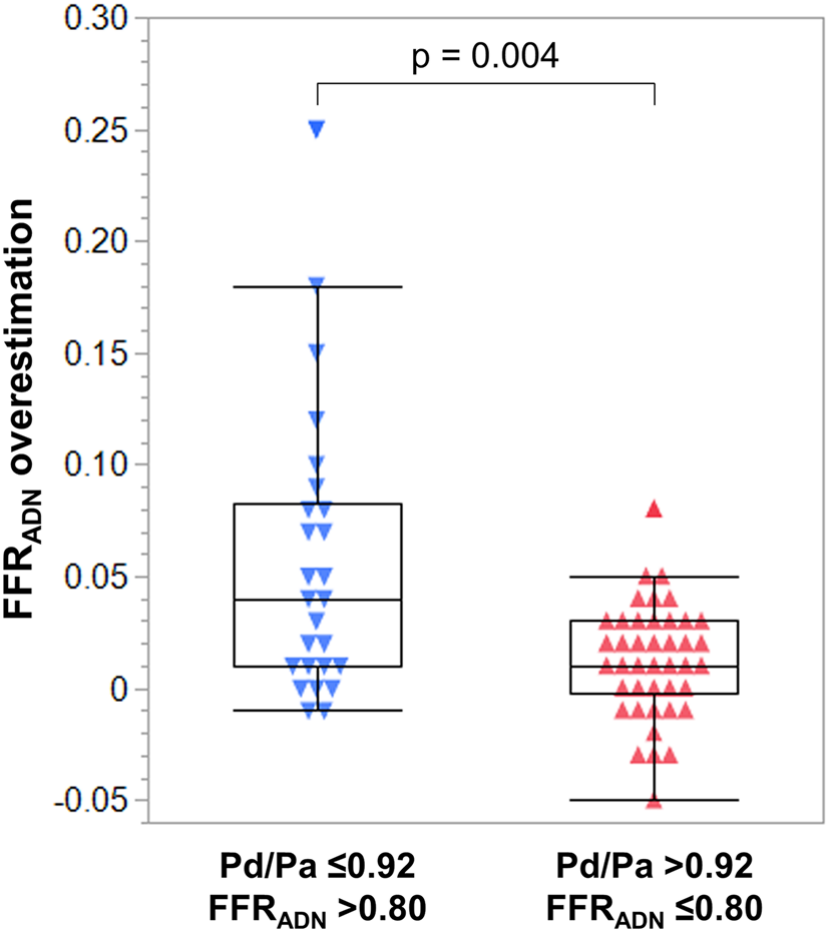
Comparison of FFR_ADN_ overestimation between low Pd/Pa–high FFR_ADN_ and high Pd/Pa–low FFR_ADN_ groups. Distributions of FFR_ADN_ overestimation with box-and-whisker plots are shown. FFR_ADN_ overestimation was defined as FFR_ADN_– FFR_PAP_. *FFR*_*ADN*_ fractional flow reserve value associated with adenosine, *FFR*_*PAP*_ fractional flow reserve value associated with papaverine, *Pd/Pa* distal-to-aortic pressure ratio.

In both of the discordance groups, adenosine resulted in higher FFR values compared to papaverine: 0.79 (IQR 0.76–0.82) vs. 0.77 (IQR 0.72–0.79), p < 0.001 for all; 0.82 (IQR 0.81–0.84) vs. 0.79 (IQR 0.74–0.81), p < 0.001 for the low Pd/Pa–high FFR_ADN_ group; and 0.77 (IQR 0.74–0.79) vs. 0.75 (IQR 0.72–0.78), p = 0.002 for the high Pd/Pa–low FFR_ADN_ group. Figure 3 demonstrates the groups’ distributions of FFR_ADN_ − FFR_PAP_. FFR_ADN_ − FFR_PAP_ was significantly greater in the low Pd/Pa–high FFR_ADN_ group compared to the high Pd/Pa–low FFR_ADN_ group: 0.04 (IQR 0.01–0.09) vs. 0.01 (IQR 0–0.03), p = 0.004. FFR_ADN_ − FFR_PAP_ ≥ 0.05 was significantly more frequently observed in the low Pd/Pa–high FFR_ADN_ group compared to the high Pd/Pa–low FFR_ADN_ group: 46% (12/26) vs. 7% (3/42), p < 0.001.

### Factors associated with the low resting Pd/Pa–high FFR_ADN_ discordance

Based on the results of the univariable analysis (Table 3A), we entered FFR_ADN_ − FFR_PAP_ (p = 0.002), diabetes mellitus (p = 0.049), hemodialysis (p = 0.080), and heart rate at baseline (p = 0.008) into the multivariable model. The multivariable logistic regression analysis identified FFR_ADN_ − FFR_PAP_ (odds ratio 1.34 per 0.01 increase, 95% confidence interval: 1.14–1.68, p = 0.002) and heart rate at baseline (odds ratio 1.07 per 1 beat/min increase, 95% confidence interval: 1.00–1.13, p = 0.048) as independent factors associated with the low resting Pd/Pa–high FFR_ADN_ discordance (Table 3B).

**Table 3:** Association with low resting Pd/Pa–high FFR_ADN_ discordance.

	Odds ratio	95% confidence interval	p value
(A) Univariable analysis			
Age (per 1 year increase)	1.04	0.98–1.10	0.222
Male	0.54	0.17–1.78	0.313
Body mass index (per 1 kg/m^2^ increase)	0.91	0.77–1.07	0.247
Hypertension	1.09	0.40–2.96	0.869
Diabetes mellitus	2.82	1.00–7.91	0.049
Dyslipidemia	1.13	0.39–3.22	0.826
Prior myocardial infarction	0.87	0.26–2.96	0.828
Prior revascularization	0.76	0.28–2.05	0.583
Current smoker	1.28	0.39–4.21	0.690
eGFR (per 1 mL/min increase)	1.00	0.98–1.02	0.877
eGFR < 60 mL/min, n (%)	1.32	0.48–3.59	0.587
Hemodialysis	7.45	0.78–70.85	0.080
Hemoglobin (per 1 g/dL increase)	1.28	0.39–4.21	0.690
LAD location	0.91	0.34–2.42	0.849
Multivessel disease	0.94	0.34–2.65	0.914
Quantitative coronary angiography			
Reference diameter (per 0.1 mm increase)	0.97	0.90–1.05	0.479
Minimal luminal diameter (per 0.1 mm increase)	1.00	0.89–1.13	0.993
Diameter stenosis (per 1% increase)	0.98	0.94–1.02	0.400
Lesion length (per 1 mm increase)	1.03	0.97–1.09	0.347
Physiologically diffuse pattern	1.47	0.54–4.02	0.457
Hemodynamic parameters			
Heart rate at baseline (per 1 beats/min increase)	1.06	1.02–1.11	0.008
Pa at baseline (per 1 mmHg increase)	0.98	0.95–1.01	0.289
FFR_ADN_ − FFR_PAP_ (per 0.01 increase)	1.33	1.14–1.64	0.002
(B) Multivariable analysis			
Diabetes mellitus	2.52	0.69–9.25	0.163
Hemodialysis	4.18	0.34–51.12	0.263
Heart rate at baseline (per 1 beats/min increase)	1.07	1.00–1.13	0.048
FFR_ADN_–FFR_PAP_ (per 0.01 increase)	1.34	1.14–1.68	0.002

Values are expressed as medians (interquartile ranges) or numbers (percentages).

*eGFR* estimated glomerular rate, *FFR*_*ADN*_ fractional flow reserve value associated with adenosine, *FFR*_*PAP*_ fractional flow reserve value associated with papaverine, *LAD* left anterior descending coronary artery, *Pd/Pa* distal-to-aortic pressure ratio.

### Reclassification of functional significance by papaverine

**Figure 4:**
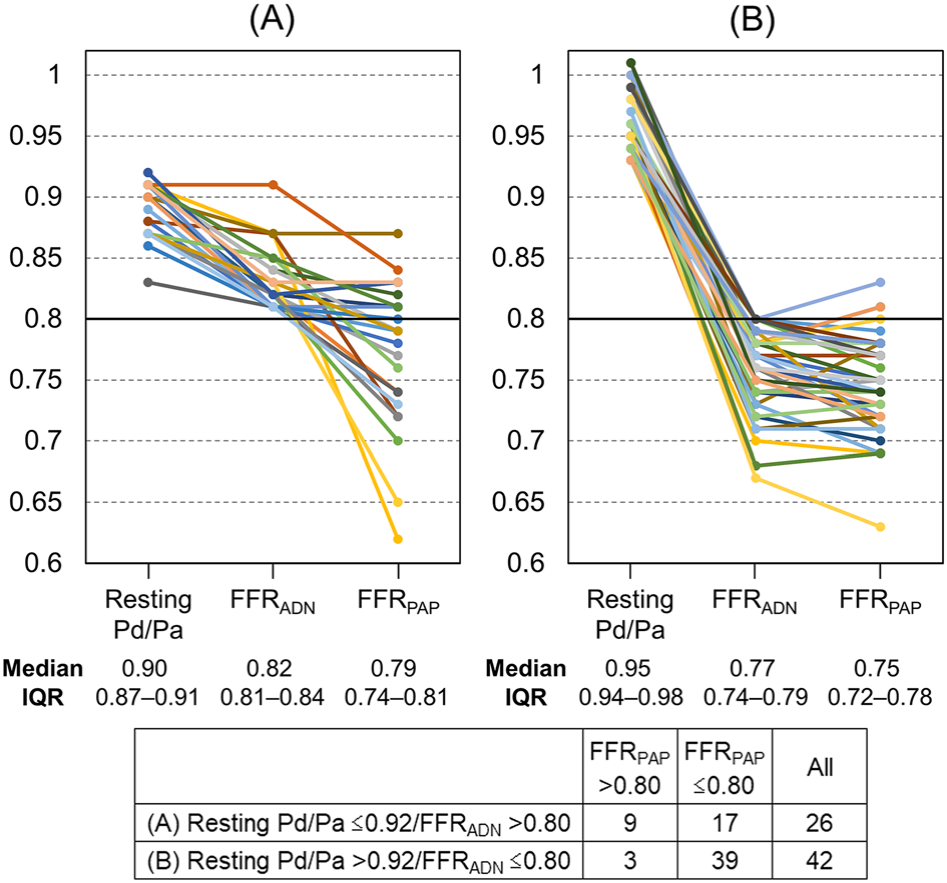
Individual resting Pd/Pa, FFR_ADN_, and FFR_PAP_ values in the low Pd/Pa − high FFR_ADN_ and high Pd/Pa − low FFR_ADN_ groups. (**A**) Low Pd/Pa − high FFR_ADN_ group (resting Pd/Pa > 0.92 and FFR_ADN_ ≤ 0.80). (**B**) High Pd/Pa − low FFR_ADN_ group (resting Pd/Pa ≤ 0.92 and FFR_ADN_ > 0.80). *FFR*_*ADN*_ fractional flow reserve value associated with adenosine, *FFR*_*PAP*_ fractional flow reserve value associated with papaverine, *IQR* interquartile range, *Pd/Pa* distal-to-aortic pressure ratio.

Figure 4 depicts individual patients’ resting Pd/Pa, FFR_ADN_, and FFR_PAP_ values. In the low Pd/Pa–high FFR_ADN_ group (Fig. 4A), papaverine produced a further decline in the FFR value in 21 vessels (81%) compared with the FFR_ADN_ value. Of the 26 vessels with the low Pd/Pa–high FFR_ADN_ discordance, the reclassification of functional significance by papaverine (FFR_ADN_ > 0.80 and FFR_PAP_ ≤ 0.80) was observed in 17 vessels (65%). Of these, 11 vessels showed FFR_ADN_ − FFR_PAP_ ≥ 0.05, and 8 had an FFR_PAP_ value below the gray zone (≤ 0.75).

Among the 42 vessels with the high Pd/Pa–low FFR_ADN_ discordance (Fig. 4B), reverse reclassification (FFR_ADN_ ≤ 0.80 and FFR_PAP_ > 0.80) was observed in only 7% (3/42) of the cases. All of the cases with reverse reclassification had a borderline FFR_ADN_ value (0.78–0.80), with a small difference from the FFR_PAP_ value (≤ 0.03).

## Discussion

Our evaluation of resting Pd/Pa and FFR measured using adenosine and papaverine revealed the following: (1) the overestimation of FFR by adenosine (i.e., insufficient adenosine-induced hyperemia) was the strongest determinant of the low Pd/Pa–high FFR_ADN_ discordance, and (2) in two-thirds of the vessels with low Pd/Pa–high FFR_ADN_ discordance, functional significance was reclassified from a negative result by adenosine (FFR_ADN_ > 0.80) to a positive result by papaverine (FFR_PAP_ ≤ 0.80). This study is first to demonstrate that insufficient adenosine-induced hyperemia is a major determinant of NHPR–FFR discordance and to clarify its influence on the reclassification of functional significance.

### Determinants of low NHPR–high FFR discordance

Due to the differences in physiologic backgrounds between resting and hyperemic conditions, the discordance between NHPR and FFR is not surprising. Coronary flow characteristics and/or microvascular resistance were demonstrated to be associated with NHPR–FFR discordance^20,21^. In vessels with preserved microvascular function (i.e., high coronary flow reserve and low microcirculatory resistance), increased coronary flow during hyperemia produces a greater pressure gradient across stenosis compared to vessels with microvascular dysfunction, leading to high NHPR–low FFR discordance. Conversely, in the presence of impaired microvascular function (i.e., low coronary flow reserve and high microcirculatory resistance), the trans-stenotic pressure gradient during hyperemia is less evident than in vessels with preserved microvascular function, leading to low NHPR–high FFR discordance.

Interestingly, we observed large overestimations of FFR by adenosine (≥ 0.05, which exceeds 2 standard deviations between repeated FFR_ADN_ measurements)^27^ in as many as 42% of the vessels with low Pd/Pa–high FFR_ADN_ discordance, but in only 7% of vessels with high Pd/Pa–low FFR_ADN_ discordance. This result suggests that the standard 140 μg/kg/min dose of intravenous adenosine may not be sufficient to induce maximal hyperemia in the presence of microvascular dysfunction. The microvascular dysfunction in vessels with low NHPR–high FFR discordance described in previous studies might be attributable in part to submaximal adenosine-induced hyperemia. Further research is necessary to address this possibility.

Insufficient adenosine-induced hyperemia due to caffeine remaining in the blood could also account for the low Pd/Pa–high FFR_ADN_ discordance. Caffeine competitively antagonizes adenosine by blocking adenosine A_2a_ receptor activity^31^. In the presence of serum caffeine, adenosine overestimated FFR in a linear concentration–response manner, compared with papaverine without involving the adenosine receptors^18^. Despite the lack of systematic pre-procedure caffeine abstinence in our present study population, the patient series reflected real-world clinical situations. Matsumoto et al. reported the associations of the duration of caffeine abstinence with serum caffeine level and FFR_ADN_ − FFR_PAP_^17^. Even after caffeine abstinence for 12–24 h, as recommended by non-invasive imaging guidelines^32,33^, serum caffeine was still detectable in most patients^17^. The mean difference between FFR_ADN_ and FFR_PAP_ (0.02) observed in the present study is similar to that after caffeine abstinence for 12–24 h^17^. More prolonged caffeine avoidance for > 48 h was shown to achieve zero serum caffeine levels in most cases and to result in comparable FFR values between adenosine and papaverine^17^; however, such strict caffeine control for all patients undergoing invasive angiography is impractical in routine care. Consequently, the frequency of low Pd/Pa–high FFR_ADN_ discordance in the present investigation was consistent with that of the low NHPR–high FFR_ADN_ discordance in earlier investigations that used adenosine or adenosine triphosphate^11,19–22^. Although it is unclear whether the patients abstained from caffeine in the prior studies^11,19–22^, caffeine antagonism might have contributed, in part, to their low NHPR–high FFR_ADN_ discordance.

Our analyses also identified the patient’s heart rate at baseline as an independent determinant of low Pd/Pa–high FFR_ADN_ discordance. This result is reasonable from a physiological point of view. The resting coronary flow increases with a higher heart rate, producing a larger resting pressure gradient^34^.

### Reclassification of functional significance

Although there is no doubt regarding the revascularization of lesions with both a low NHPR and a low FFR, it remains unclear whether or not lesions with NHPR–FFR discordance should be revascularized. Lee et al. reported that major adverse cardiovascular events were increased only when both NHPR and FFR were positive^35^. Notably, in two-thirds of the present cases of low Pd/Pa–high FFR_ADN_ discordance, the physiological significance was reclassified from a negative result by adenosine to a positive result by papaverine; that is, false-negative FFR results were provided by adenosine. In addition, two-thirds of these false-negative adenosine-induced FFR results were attributed to a large overestimation of FFR by adenosine, i.e., ≥ 0.05. Patients with false-negative results that are due specifically to large overestimations of FFR miss the opportunity to receive benefits from revascularization, which may lead to adverse outcomes. Other investigations have indicated that when the patients are treated with medical therapy alone, their FFR values, even around the cut-off value, demonstrated a continuous relationship with subsequent adverse coronary events^36,37^.

Based on landmark FFR studies (DEFER, FAME I, and FAME II) in which mainly intravenous adenosine was used for hyperemia induction^1,2,38^, the rate of major adverse cardiac events in deferred lesions was considered to be approximately 1% per year^3^. In a recent large-scale prospective observational trial (the J-CONFIRM registry), major adverse cardiac events occurred less frequently, in as few as 0.4% of deferred lesions^39^. Although none of the reports of these trials provided information on serum caffeine levels or the length of caffeine abstinence, the lower incidence of major adverse cardiac events in the J-CONFIRM trial might have occurred in part because hyperemic stimuli other than adenosine (e.g., papaverine or nicorandil) that do not involve the adenosine receptors were used in more than half of their study patients^39^. Further investigation is necessary to confirm the prognostic values of papaverine- and nicorandil-induced FFR.

Insufficient adenosine-induced hyperemia and/or reclassification of functional significance will not be identified unless another hyperemic stimulus is used. Given the present high incidence (two-thirds) of false-negative FFR_ADN_ results, low NHPR–high FFR_ADN_ discordance mismatch may alert operators to insufficient adenosine-induced hyperemia. In the presence of low NHPR–high FFR_ADN_ discordance, the use of other hyperemic stimuli that do not involve the adenosine A_2a_ receptors (e.g., papaverine and nicorandil) should be considered to avoid misinterpretations of physiological significance.

Instead of wire-derived physiological indices, wire-free angiography-derived computational indices of FFR, such as quantitative flow ratio, have been introduced^40^. Quantitative flow ratio was also reported to show discordance with FFR^41^. Considering that both NHPRs and quantitative flow ratio are measured under non-hyperemic conditions, insufficient hyperemia would cause low quantitative flow ratio–high FFR discordance. In other words, all non-hyperemic physiological indices may provide a clue about insufficient hyperemia.

### Study limitations

Several limitations should be acknowledged. First, the number of cases of low Pd/Pa–high FFR_ADN_ discordance was relatively small. Second, the prognostic relevance of low Pd/Pa–high FFR_ADN_ discordance and/or reclassification could not be identified in this study, because some of the vessels with low Pd/Pa–high FFR_ADN_ discordance were revascularized based on positive FFR_PAP_ (≤ 0.80) results. Further research is warranted to address whether NHPR–FFR discordance due to insufficient adenosine-induced hyperemia is associated with adverse outcomes. Third, microvascular function was not assessed. Microcirculatory resistance cannot be accurately evaluated by adenosine in the presence of insufficient adenosine-induced hyperemia. Lastly, the order of hyperemic agents was fixed (papaverine last) because papaverine was used to obtain a reliable pullback curve. Although papaverine was administered after confirming that Pd/Pa values had returned to the baseline level, adenosine’s carry-over effect cannot be excluded.

## Conclusions

Insufficient adenosine-induced hyperemia is a major determinant of the low resting Pd/Pa–high FFR discordance. Physicians should bear in mind that the presence of a low non-hyperemic pressure ratio but a high adenosine-induced FFR may indicate a false-negative FFR result.

## Data Availability

The data that support the findings of this study are available from the corresponding author upon reasonable request.

## Abbreviations

FFR: Fractional flow reserve
FFR_ADN_: Fractional flow reserve value associated with adenosine
FFR_PAP_: Fractional flow reserve value associated with papaverine
IQR: Interquartile range
LAD: Left anterior descending coronary artery
NHPR: Non-hyperemic pressure ratio
Pa: Aortic pressure
Pd: Distal coronary pressure
Pd/Pa: Distal-to-aortic pressure ratio
